# Impact of the COVID-19 Pandemic on Patients and Staff in Radiation Oncology Departments in Belgium: A National Survey

**DOI:** 10.3389/fonc.2021.654086

**Published:** 2021-03-19

**Authors:** Aude Vaandering, Selma Ben Mustapha, Maarten Lambrecht, Dirk Van Gestel, Liv Veldmeman

**Affiliations:** ^1^Radiation Oncology Department, Cliniques Universitaires Saint Luc, Brussels, Belgium; ^2^Center of Molecular Imaging, Radiotherapy and Oncology (MIRO), Institut de Recherche Expérimentale et Clinique (IREC), Université catholique de Louvain, Brussels, Belgium; ^3^Department of Radiation Oncology, Centre Hospitalier Universitaire (CHU) de Liège, University of Liège, Liège, Belgium; ^4^Department of Radiotherapy-Oncology, Leuven Kanker Instituut, Universitair Ziekenhuis (UZ) Gasthuisberg, Leuven, Belgium; ^5^Department of Radiation Oncology, Institut Jules Bordet, Université Libre de Bruxelles, Brussels, Belgium; ^6^Department of Human Structure and Repair, Ghent University, Ghent, Belgium; ^7^Department of Radiation Oncology, Ghent University Hospital, Ghent, Belgium

**Keywords:** COVID-19, radiotherapy, radiation oncology, national survey, pandemic

## Abstract

**Purpose:** COVID-19 reached Belgium in February and quickly became a major public health challenge. It is of importance to evaluate the actual impact of COVID-19 on patients and staff in Belgian radiotherapy departments (RTDs). This was evaluated through a weekly national survey sent to departments measuring key factors that were affected by the pandemic.

**Materials and Methods:** The Belgian SocieTy for Radiation Oncology (BeSTRO) together with the Belgian College for physicians in Radiation Oncology invited all 26 RTD to participate in a survey that started on March 2nd and was re- submitted weekly for 4 months to assess variations in time. The survey focused on: (1) the COVID-19 status of patients and staff; (2) the management of clinically suspected COVID patients and COVID positive patients; (3) the impact of COVID-19 on RTD activities; (4) its impact in radiotherapy indications and fractionation schemes.

**Results:** Seventy-three percent of 26 RTDs completed the first survey and 57% responded to all weekly surveys. In the RTD staff, 24 members were COVID-positive of whom 67% were RTTs. Over the study period, the number of patients treated dropped by a maximum of 18.8% when compared to March 2nd. In 32.3% of COVID-positive and 54% of COVID suspected patients, treatment was continued without any interruptions. Radiotherapy indications were adapted within the 1st weeks of the survey in 47.4% of RTD, especially for urological and breast tumors. Fractionation schemes were changed in 68.4% of RTD, mainly for urological, breast, gastro-intestinal, and lung tumors.

**Conclusions:** Between March and June 2020, the COVID-19 pandemic resulted in an important decrease in treatment activity in RTD in Belgium (18.8%). The COVID-19 infection status of patients influenced the continuity of the radiotherapy schedule. Changes in indications and fractionation schedules of radiotherapy were rapidly incorporated in the different RTD.

## Introduction

In December 2019, the Chinese authorities alerted the World Health Organization (WHO) that a cluster of pneumonia cases of unknown origin had been detected. The cause was quickly identified as being a novel coronavirus tagged as SARS-CoV-2 (COVID-19) (1). Since then, the epidemic has become a pandemic with the global spread of COVID-19 leading to RTD having to reorganize and reschedule their daily throughput in accordance with government and institutional recommendations. In parallel, national, American, and European scientific societies have issued recommendations to guide RTD in the management of certain types of cancer (2–4).

Many publications from severely touched COVID-19 regions like China (5), Iran (6), and Italy (7, 8) also shared the techniques and strategies they set up to help ensure patient and staff safety in the context of the pandemic.

On behalf of the Belgian SocieTy for Radiation Oncology (BeSTRO) and the Belgian College for Physicians in Radiation Oncology, we decided that it was important to monitor the impact of the COVID pandemic on RTD and their treatment activities. This was realized through a weekly survey that was sent out to all departments.

This article presents the results of this multicentric Belgian radiotherapy data collection project, which includes monitoring the status of COVID testing for patients and staff, the impact of the pandemic on the number of treatments, the management of clinically suspected and COVID positive patients and the actual changes made to radiotherapy treatment indications and/or fractionation schemes.

## Materials and Methods

### Survey Design and Data Collection

A 25-question survey was designed to be addressed to the 24 primary RTD in Belgium to investigate how they adapted to the COVID-19 pandemic. Two satellite sites were also independently contacted as their treatment data is separate from their main site. The questionnaire was designated in order to collect the following items:

- Covid-19 testing of RTD staff and patients
- Impact of COVID-19 on the number of radiotherapy treatments
- Impact of patient COVID-19 status on treatment delivery
- Changes in radiotherapy indications and fractionation schemes.

The complete questionnaire can be found in Appendix A (Supplementary Material). The questionnaire was built by the authors according to the published recommendations (5, 9, 10). In order to assess the validity and appropriateness of the survey, the first draft was sent to a panel of radiation oncologists (co-authors) from different institutions. Their remarks and proposals were incorporated within the final survey. Only one answer per contacted RTD was allowed to avoid a data redundancy from a single institution.

The initial survey (Appendix A in Supplementary Material) was sent on April 14th 2020 in order to capture data from the week of March 2nd till the week of April 6th. A weekly follow up survey was then sent from the week of April 13th till June 22nd included in which the department was asked to complete the data for the week in question. Data was collected and managed using REDCap electronic data capture tools hosted at the Cliniques Universitaires St Luc. REDCap (Research Electronic Data Capture) is a secure, web-based application designed to support data capture for research studies (11, 12).

Since the investigation didn't focus on patients or animals and no clinical files were considered, the endorsement of the ethics committee was not requested.

All the answers to the surveys were gathered from March 2nd until June 26th included. The survey was closed in June 2020 as the response rate was dropping and as the first COVID-19 wave died out.

### Data Analysis

The collected data was exported from RedCap into a Microsoft Excel file (2018) and IBM SPSS Statistics for Windows, Version 26. Descriptive statistical analysis was carried out in both systems. Data extraction was carried out beginning of April and at the end of the data collection period. This allowed us to re-contact departments if data needed to be reviewed. But this also allowed us to give feedback to departments by sending them personalized reports in which they could visualize the data that was collected in their own department in comparison to national trends.

For the analysis of the departments' treatment activity levels, the number of treatments recorded for the 1st week of the survey (week of March 2nd) was set as the baseline to which the following weeks were compared to.

## Results

Twenty-one of the 26 RTD responded of which 19 departments completed the initial survey (73% participation rate). The number of respondents dropped over time with 15 departments completing the survey during the last week.

Among the 19 departments that responded were all 7 academic hospitals RTD (36.8%).

The mean number of Linacs per department was 3.4 with a minimum of 1 and a maximum of 6 Linacs.

**Figure 1:**
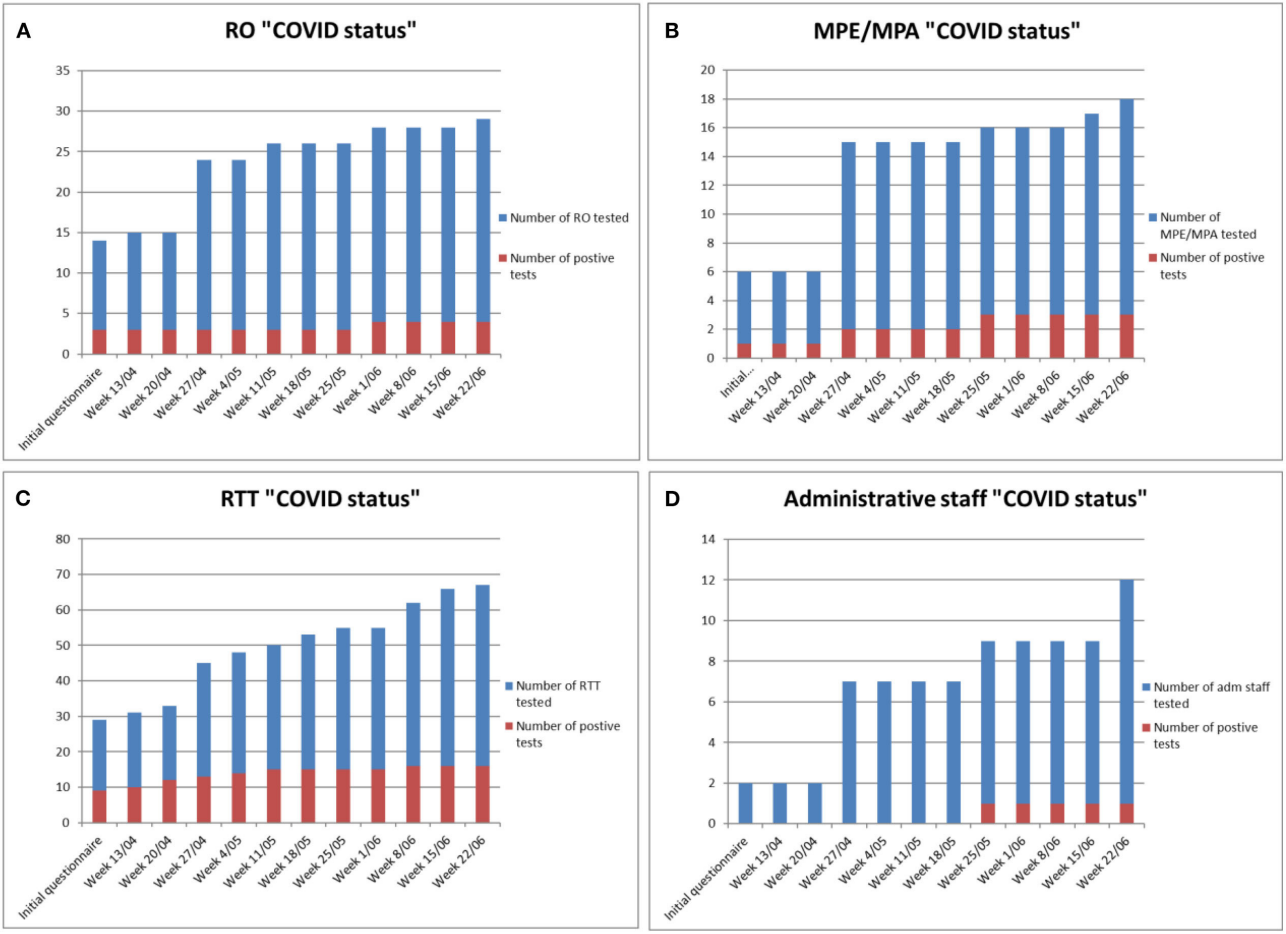
Cumulative number of RTD staff members tested and number of staff tested positive for COVID-19: for **(A)** Radiation Oncologists (RO); **(B)** Medical Physics Experts (MPE) and Medical Physics Assistants (MPA); **(C)** Radiation therapists (RTTs) and **(D)** Administrative staff.

The number of RTD staff members getting tested per week increased for all members of staff during the studied period, however the positivity rate seemed to stay quite low (Figure 1).

At the end of the study period, overall, among 129 tested staff members, 24 were confirmed COVID-positive. This represents a positivity rate of 13.8, 16.6, 23.8, and 8.3% Radiation Oncologists (RO), Medical physics experts/medial physics assistants (MPE/MPA), radiation therapists (RTT) and administrative staff, respectively. Based on the 2019 data collected in the framework of a national quality indicator project which, amongst others, collects national staffing level in RTD, this would amount to 3% of ROs being infected, 2% of MPEs & MPAs being infected, and 4% of RTTs being infected.

**Figure 2:**
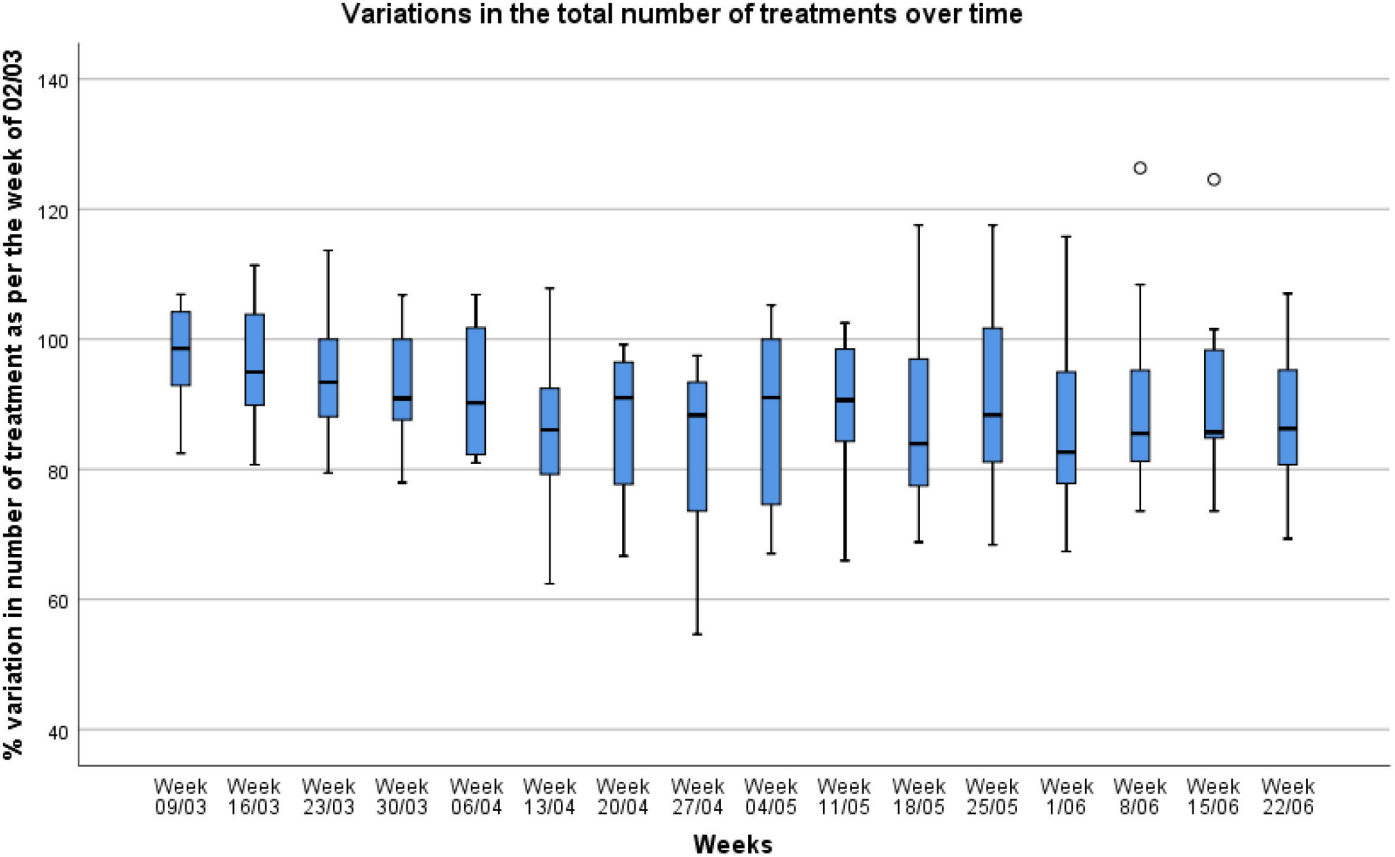
Variations in the total number of treatments between March 9th and June 26th (assuming that the week of March 2nd is a week where there was a 100% activity level).

The number of patients being tested increased from 124 patients in the initial questionnaire to 603 in the last week. Ninety patients were tested COVID-positive (15% positivity rate). The overall percentages of radiotherapy treatments decreased over the weeks (Figure 2) with a mean drop of 18.8% (Range: 2.5–45.4%) in the total number of treatments during the week of April 27th as compared to the number of treatments recorded for the week of March 2nd.

**Figure 3:**
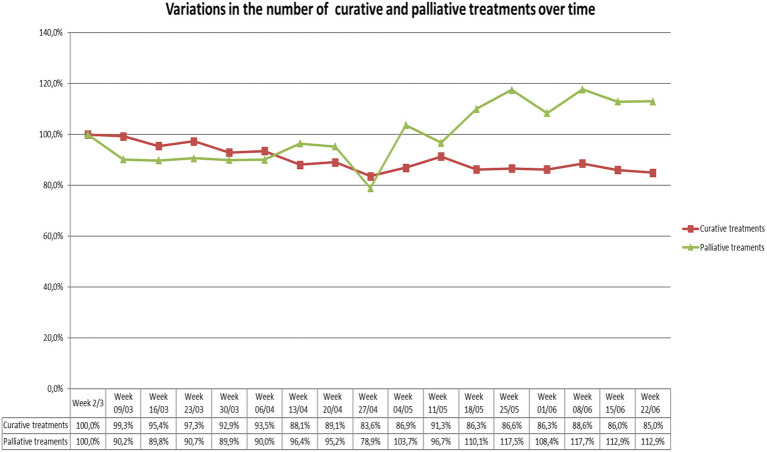
Variations in the number of curative and palliative treatments over the studied period as compared to the week of March 2nd.

During the studied period, there seems to be a decrease in the number of both curative and palliative treatments until the end of April 2020—at which time there is a sudden increase in the number of palliative treatments up to 18.2% as compared to the baseline week (Figure 3).

**Figure 4:**
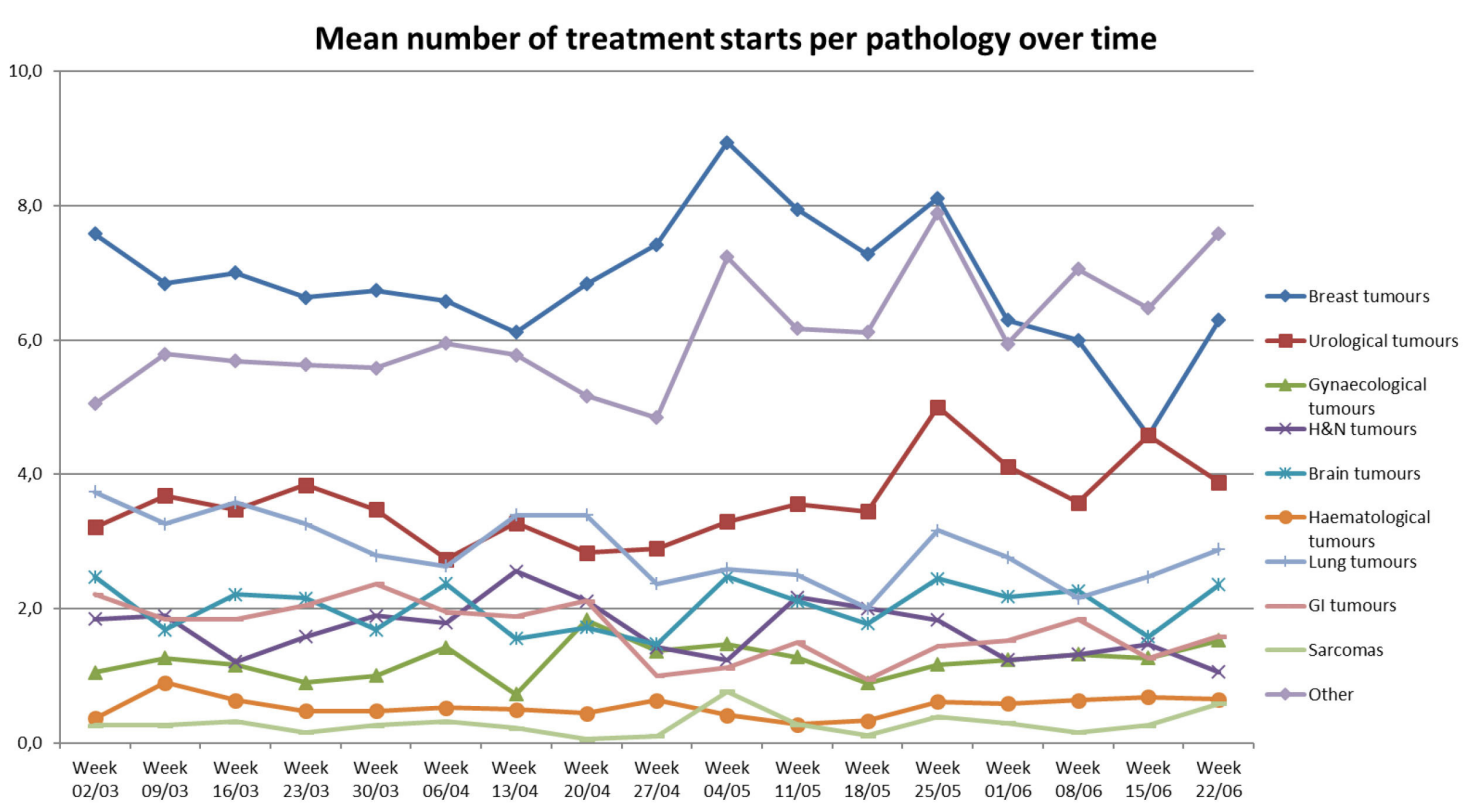
Mean number of treatment starts per department per pathology over time.

The number of treatment starts per pathology was also collected over the studied period (Figure 4). The data collected seems to illustrate a drop in the number of treatment starts for all pathologies. More particularly, there was a drop in the number of treatment starts for lung, GI and “other” tumors from the week of April 20th until the week of May 18th. The other pathology groups show variations in activity over time. However, an increase in activity can be observed during the week of May 25th, in which the number of treatment starts for breast, urological, lung, brain, GI and “other” tumors suddenly increases.

The impact of COVID-19 on the use of treatment modalities (LINACS) showed that 18 out of the 19 departments that responded, kept all their LINACS active. One department informed the authors that part of their equipment was dedicated to COVID positive patients.

**Figure 5:**
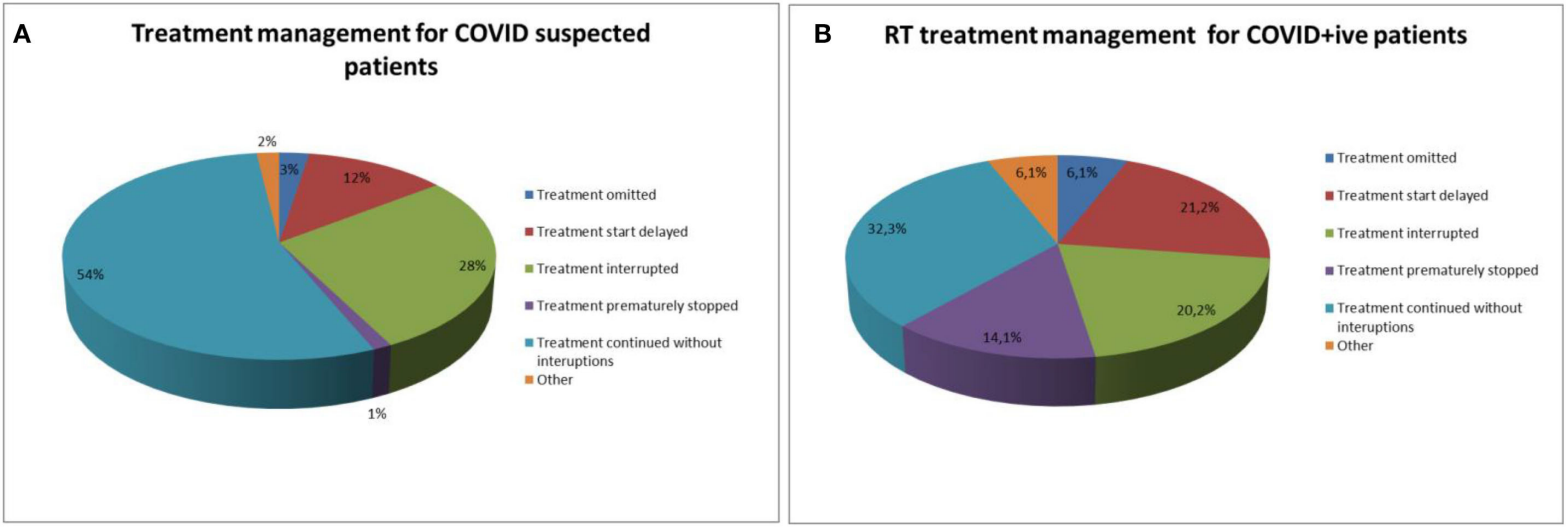
**(A)** Treatment management for COVID suspected patients. **(B)** Treatment management for COVID positive patients.

For COVID positive patients, treatment was delivered as planned in 32.3% of the cases with no interruptions in the treatment delivery process. In ~20% of the positive cases, the treatment was either delayed or interrupted. In 14% of COVID-positive patients, the treatment was prematurely stopped and in 6% of patients another element impacted the treatment (this includes patient death, patient's decision to stop their treatment or necessity to replan the treatment on a COVID-dedicated LINAC). For clinically suspected COVID-positive patients (patients presenting COVID-19-like symptoms), treatment continued as planned in 54% of patients while treatment was interrupted in 28% of patient and delayed in 12% of patients (Figures 5A,B).

**Figure 6:**
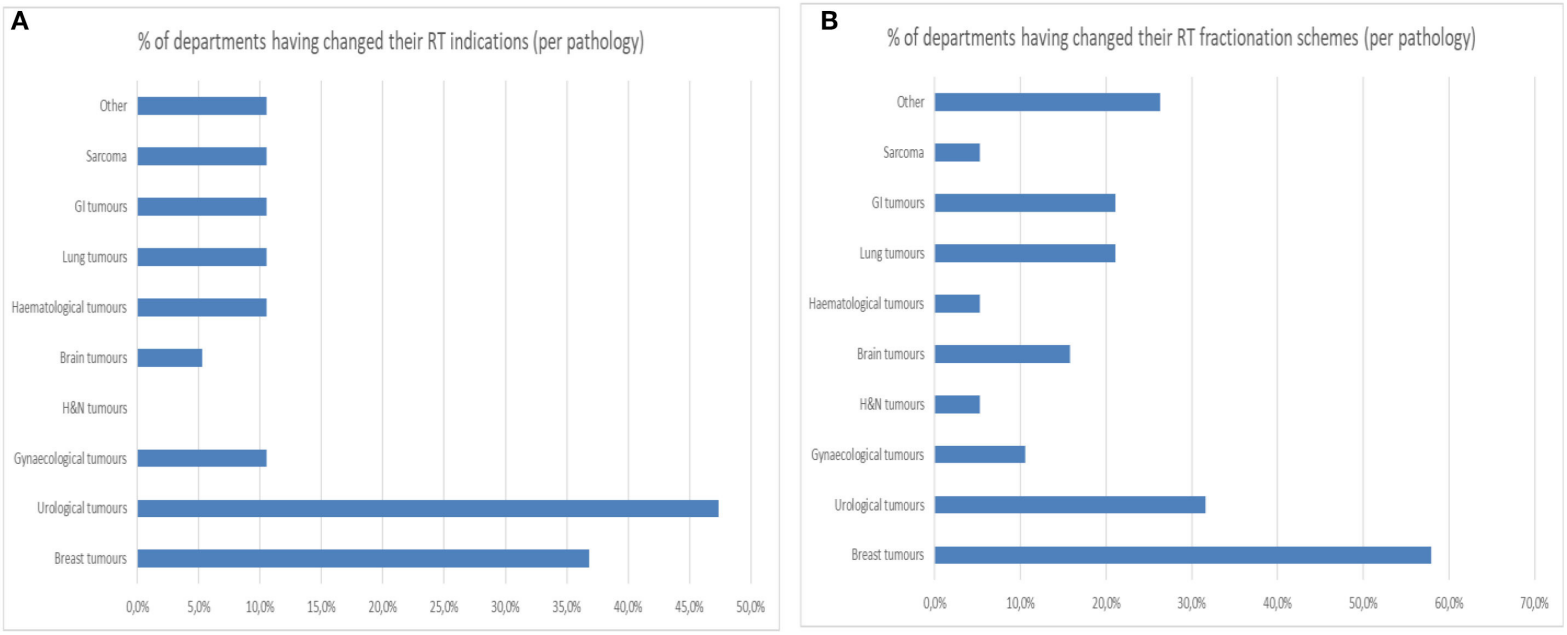
**(A)** Percentage of departments having changed their radiotherapy indication per pathology. **(B)** Percentage of departments having changed their radiotherapy fractionation schemes per pathology.

From the survey, it appears that roughly half of the departments changed some of their radiotherapy treatment indications. Indeed, 47.4% of departments changed their indication for breast tumors. Thirty-seven percent of departments changed their indication for urological tumors and 10% of departments changed their indication for gynecological, gastro-intestinal (GI), hematological, lung tumors and sarcomas. Sixty-eight percent of RTD made changes in their fractionation protocols with 31.6% of departments making these changes for urological tumors and 57.9% of RTD making these changes for breast tumors (Figures 6A,B).

## Discussions

This survey shows the impact of the COVID-19 pandemic on RTD in Belgium while also assessing their reorganization. To our knowledge, this is the first survey composed of weekly follow-ups that monitor the situation during ~4 months.

As seen in other national surveys; the number of radiotherapy treatments, in Belgium, dropped by a maximum of 18.8% (at week 8—last week of April). This is not unlike a study carried out in the USA where 55% of respondents reported a decrease in between 20 and 39% of the total number of patient volume (13), and in Switzerland where 45% of respondents reported <20% of daily activity reduction (14). This is also in line with the recent publication of the Belgian Cancer Registry (BCR), which reports a 50% decrease in the number of cancer diagnosis in April 2020 (as compared to 2019). Although, this was followed up by a catch-up phase (significant increase in number of diagnosis) during the summer of 2020, the BCR still reports a difference of 14% of reported cancer diagnosis between the 2019 and 2020 March-September period (15). This drop in cancer diagnosis and radiotherapy treatments is indeed not surprising, as the country has been thoroughly hit by the pandemic with more than 100 deaths per day observed between March 25 and April 29th 2020 (for 11.5 million inhabitants) (16). The Belgian government declared a lockdown for the entire country on March 18th and imposed all hospitals to suspend all consultations and elective activities until May 4th and 11th, respectively (17).

Most publications measuring the impact of COVID-19 on RTD are based on a single questionnaire (8, 13, 14, 18, 19). Our survey, which took place over several weeks, allows for the evaluation of the impact of the pandemic on RTH treatment over time and as such illustrates the dynamic nature of how COVID-19 impacted treatment activity. It was observed that the number of curative treatments decreased until the end of April and then started to increase again without regaining a 100% activity as compared to the reference week of March 2nd. However, the evolution of the number of palliative treatments demonstrated a significant increase in the beginning of May with an overall increase in the number of treatments of 118% as compared to the week of March 2nd. One explanation for the important increase in palliative treatment could be a catch up of primary delayed patients or patients that didn't respond to pain killers and other symptomatic medications used in a first attempt to avoid radiotherapy treatment.

The analysis of the survey also demonstrated that RTD staff was not sheltered from the COVID-19 outbreak in which the RTT staff seemed to be the most impacted. But, when carrying out the comparison of proportions, the differences between the rates of positivity to COVID testing between the different groups of staff members, where not significant. Moreover, it is important to note that not all departments were able to report the exact number of staff being tested.

When analyzing the COVID status of RT patients, the data collected indicate a positivity rate of 15% among tested patients. However, it is important to note that the rate of COVID-19 testing among RTD patients, could vary quite significantly among different hospitals and were also highly impacted by local recommendations and policies.

There was also, as observed in Lombardy (7), differences in radiotherapy management of COVID-positive and COVID suspected patients whereby treatment was started and delivered as planned in 54% of COVID-suspected patients and only 32.3% of COVID-positive patients. These differences in management probably reflect the notion of having to maintain a balance between the risk of infecting other patients or staff members and the potential harm of delaying and/or interrupting radiotherapy on oncological outcome as was shown for certain types of cancer (20).

Finally, as seen in other publications (8, 14), the data collected in the survey have put forth that a significant number of RTD have made several adjustments in their indications of radiotherapy in which 47.4% of departments adapted their indications for urological and breast tumors. Changes in fractionation protocols were applied mainly for urological, breast, GI and lung tumors as observed in Switzerland (14) where there was a 18% increase of hypofractionation use for breast radiotherapy and 23% increase of neoadjuvant short course radiotherapy for rectal cancer during the month of April 2020. The changes applied to the above mentioned pathologies was probably “facilitated” by the existing evidence for hypofractionation schemes that were later published as recommendations for COVID pandemic adaptation (21–23).

We acknowledge that there are several limitations to our study.

Firstly, the survey contained a limited number of questions and most of the questions remained quite general. This was purposely done in order to increase the probability in which departments would respond to the survey on a weekly basis and this during 4 months.

Secondly, it is important to note that the thorough analysis of the data was limited by the fact that all treatment activities were compared to the reference week of March 2nd which only represents a snapshot of departments' treatment activities. We assumed that, at that time, the COVID pandemic had not yet had an impact on radiotherapy treatments. It might be possible that the week of March 2nd already had been subjected to a decrease in activity and that our study therefore underestimates the rate of decrease in treatment activity.

Third, as previously mentioned, the rates and policies of testing were very different from one department to another and some respondents acknowledged difficulties of reporting test results especially among RTD staff for medical confidentiality issues. As such, this study may be underestimating the rates of testing and of COVID-positivity.

Furthermore, there was variation in the data reported as some RTD reported the number of patients tested only during the treatment delivery process while other departments also reported the number of tests carried out for patients in the pretreatment phase (patients who had not started their treatment yet).

## Conclusions

Between March and June 2020, the COVID pandemic impacted radiotherapy treatments in Belgium, mainly through a resulting decrease in treatment activity during the 1st months. Impact on staffing was limited. The management of patients who were COVID-19 positive or clinically suspected COVID-19 positive was also affected with treatment interruptions or delays in 68 and 46% of patients, respectively. Departments were rapid in implementing adaptations in treatment indications and fractionation schedules.

## Data Availability Statement

The raw data supporting the conclusions of this article will be made available by the authors, without undue reservation.

## Ethics Statement

Ethical review and approval was not required for the study on human participants in accordance with the local legislation and institutional requirements. Written informed consent for participation was not required for this study in accordance with the national legislation and the institutional requirements.

## Author Contributions

All authors listed have made a substantial, direct and intellectual contribution to the work, and approved it for publication.

## Conflict of Interest

The authors declare that the research was conducted in the absence of any commercial or financial relationships that could be construed as a potential conflict of interest.
