# Supplementary material for: Impact of the COVID-19 Pandemic on Patients and Staff in Radiation Oncology Departments in Belgium: A National Survey
Source: Front Oncol. 2021 Mar 19;11:654086. doi: 10.3389/fonc.2021.654086 (PMC8017334; doi:10.3389/fonc.2021.654086)
Supplement: Supplementary file 1 [file Data_Sheet_1.PDF]

Initial COVID-19 and RT cancer care survey

Welcome to this initial COVID-19 and RT cancer care survey.

Please complete the survey below.

We would like to thank you for your effort in providing this useful information.

DEPARTMENT HUMAN RESOURCES

Up till now, have any employees in your department been tested for COVID-19?

Radiation Oncologists
- ◯ Unknown
- ◯ No
- ◯ Yes

How many were tested positive?

____
(If unknown or not shareable, please type 999)

How many were tested negative?

____
(If unknown or not shareable, please type 999)

Medical physicists or dosimetrists?
- ◯ Unknown
- ◯ No
- ◯ Yes

How many were tested positive?

____
(If unknown or not shareable, please type 999)

How many were tested negative?

____
(If unknown or not shareable, please type 999)

RTTs?
- ◯ Unknown
- ◯ No
- ◯ Yes

How many were tested positive?

____
(If unknown or not shareable, please type 999)

How many were tested negative?

____
(If unknown or not shareable, please type 999)

Administrative staff?

- ◯ Unknown
- ◯ No
- ◯ Yes

How many were tested positive?

____
(If unknown or not shareable, please type 999)

How many were tested negative?

____
(If unknown or not shareable, please type 999)

Other staff?

- ◯ Unknown
- ◯ No
- ◯ Yes

How many were tested positive?

____
(If unknown or not shareable, please type 999)

How many were tested negative?

____
(If unknown or not shareable, please type 999)

COVID-19 IN PATIENTS

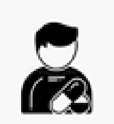

Up till now, how many patients were tested for COVID-19?

Number of patients that were tested for COVID-19

____
(If unknown or not shareable, please type 999)

Number of patients that were tested positive

____
(If unknown or not shareable, please type 999)

Number of patients that were tested negative

____
(If unknown or not shareable, please type 999)

Please specify the age and gender of all patients who tested positive.

____

For how many of those patients who tested POSTIVE for COVID-19 was radiotherapy ...

ommited?

____
(If unknown or not shareable, please type 999)

start delayed?

____
(If unknown or not shareable, please type 999)

interrupted during radiotherapy?

____
(If unknown or not shareable, please type 999)

prematurely stopped?

____
(If unknown or not shareable, please type 999)

continued without interuptions?

____
(If unknown or not shareable, please type 999)

another intervention needed?

◯ Unknown
◯ No
◯ Yes

Please explain

____

Up till now, for how many of CLINICALLY SUSPECTED COVID-19 +ive patients (either not tested or tested negative) was radiotherapy ...

omitted?

____
(If unknown or not shareable, please type 999)

start delayed?

____
(If unknown or not shareable, please type 999)

interrupted?

____
(If unknown or not shareable, please type 999)

prematurely stopped?

____
(If unknown or not shareable, please type 999)

continued without interuptions?

____
(If unknown or not shareable, please type 999)

another intervention?

◯ Unknown
◯ No
◯ Yes

Please specify

____

Were specific measures taken during RT for clinically suspected COVID +ive patients?

◯ Unknown
◯ No
◯ Yes
◯ N/A

Were specific measures taken during RT for COVID-19 positive patients?

◯ Unknown
◯ No
◯ Yes
◯ N/A

RADIOTHERAPY DEPARTMENT ACTIVITY

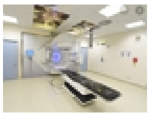

How many LINACs are still operational?

____

How many patients did you treat with CURATIVE intent...
(total number of patients)

during the week of 2/3?

____

during the week of 09/3?

____

during the week of 16/3?

____

during the week of 23/3?

____

during the week of 30/3?

____

during last week (06/04)?

____

How many patients did you treat with PALLIATIVE intent ...
(total number of patients)

during the week of 2/3?

____

during the week of 09/3?

____

during the week of 16/3?

____

during the week of 23/3? __________________________________

during the week of 30/3? __________________________________

during last week (06/04)? __________________________________

How many patients started their treatment (first fraction) during the week of 2/3 for the following tumours?

breast tumours __________________________________

urological tumours __________________________________

gynaecological tumours __________________________________

head and neck tumours __________________________________

brain tumours __________________________________

hematological malignancies __________________________________

lung tumours __________________________________

gastro-intestinal tumours __________________________________

sarcomas __________________________________

other pathologies __________________________________

How many patients started their treatment (first fraction) during the week of 10/3 for the following tumours?

breast tumours __________________________________

urological tumours __________________________________

gynaecological tumours __________________________________

head and neck tumours ______________________

brain tumours ______________________

haematological malignancies ______________________

lung tumours ______________________

gastro-intestinal tumours ______________________

sarcomas ______________________

other pathologies ______________________

How many patients started their treatment (first fraction) during the week of 17/3 for the following tumours?

breast tumours ______________________

urological tumours ______________________

gynaecological tumours ______________________

head and neck tumours ______________________

brain tumours ______________________

haematological malignancies ______________________

lung tumours ______________________

gastro-intestinal tumours ______________________

sarcomas ______________________

other pathologies ______________________

How many patients started their treatment (first fraction) during the week of 24/3 for the following tumours?

breast tumours ______________________

urological tumours ______________________

gynaecological tumours ______________________

head and neck tumours ______________________

brain tumours ______________________

haematological malignancies ______________________

lung tumours ______________________

gastro-intestinal tumours ______________________

sarcomas ______________________

other pathologies ______________________

How many patients started their treatment (first fraction) during the week of 31/3 for the following tumours?

breast tumours ______________________

urological tumours ______________________

gynaecological tumours ______________________

head and neck tumours ______________________

brain tumours ______________________

haematological malignancies ______________________

lung tumours ____________________

gastro-intestinal tumours ____________________

sarcomas ____________________

other pathologies ____________________

How many patients started their treatment (first fraction) during last week (week 6/04)?

breast tumours ____________________

urological tumours ____________________

gynaecological tumours ____________________

head and neck tumours ____________________

brain tumours ____________________

haematological malignancies ____________________

lung tumours ____________________

gastro-intestinal tumours ____________________

sarcomas ____________________

other pathologies ____________________

RADIOTHERAPY PRACTICE

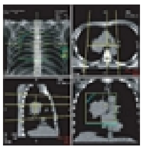

Did you make any changes in the indications for radiotherapy?

◯ Yes
◯ No
(e.g. no radiotherapy for T1N0M0 ER+ breast cancer patients of older age)

For which pathologies?	☐ breast tumours<br>☐ urological tumours<br>☐ gynaecological tumours<br>☐ head and neck tumours<br>☐ brain tumours<br>☐ haematological malignancies<br>☐ lung tumours<br>☐ gastro-intestinal tumours<br>☐ sarcomas<br>☐ other pathologies
Did you make any changes in fractionation schedules?	◯ Yes<br>◯ No
For which pathologies?	☐ breast tumours<br>☐ urological tumours<br>☐ gynaecological tumours<br>☐ head and neck tumours<br>☐ brain tumours<br>☐ haematological malignancies<br>☐ lung tumours<br>☐ gastro-intestinal tumours<br>☐ sarcomas<br>☐ other pathologies
